# Supplementary material for: Optimised weight programming for analogue memory-based deep neural networks
Source: Nat Commun. 2022 Jun 30;13:3765. doi: 10.1038/s41467-022-31405-1 (PMC9247051; doi:10.1038/s41467-022-31405-1)
Supplement: Supplementary file 1 — Supplementary Information [file 41467_2022_31405_MOESM1_ESM.pdf]

Supplementary Information

Charles Mackin[1], Malte Rasch[2], An Chen[1], Jonathan Timcheck[3], Robert L. Bruce[2], Ning Li[2], Pritish Narayanan[1], Stefano Ambrogio[1], Manuel Le Gallo[4], S. R. Nandakumar[4], Andrea Fasoli[1], Jose Luquin[1], Alexander Friz[1], Abu Sebastian[4], Hsinyu Tsai[1] & Geoffrey W. Burr[1]

[1]*IBM Research–Almaden, San Jose, CA, USA*

[2]*IBM Research–Yorktown, Yorktown Heights, NY, USA*

[3]*Stanford University, Stanford, CA, USA*

[4]*IBM Research–Zürich, Switzerland*

Hardware-Aware Training Hyper-parameter Scan

The following shows hardware-aware (HWA) training results for the LSTM, ResNet-32, and BERT-base networks referenced in this work. A number of hyper-parameters were scanned to achieve the best possible accuracies. In each case, HWA trained networks reach 32-bit floating-point (FP32) equivalent (or near equivalent) accuracy. FP32 training results, which represent conventionally trained DNNs, serve as the target baseline. Weight noise, with similar characteristics to weight programming noise, and read noise are added during HWA training along other hardware nonidealities (see Methods). The normalised level of PCM weight noise is swept using the 'rpu_hwa_weight_noise_std_dev' parameter. You will note that this parameter may vary from 0.0 (no weight noise) to 5.0 (meaning five times greater weight noise than what is expected from our characterised PCM devices).

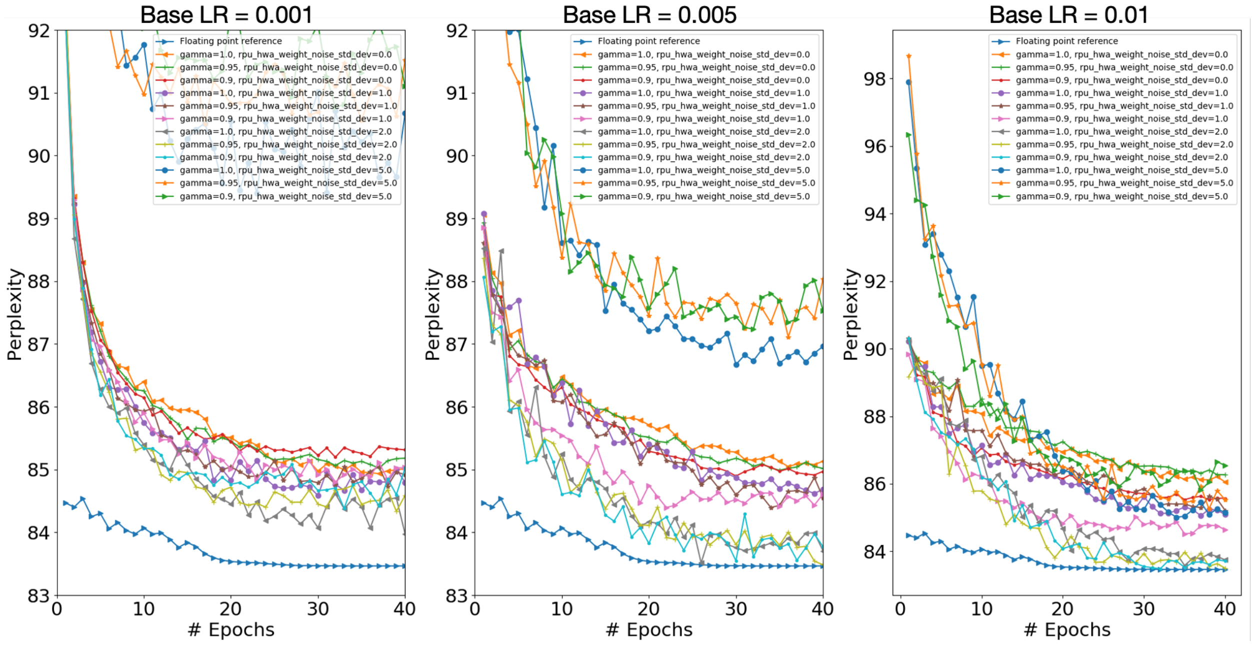

Supplementary Figure 1: Hyper-parameter scan for the hardware-aware training of the two-layer LSTM network on the Penn Tree Bank dataset. Plots depict some of the hyper-parameters scanned while optimising the hardware-aware training, which include the base learning rate, learning rate decay factor gamma, and the normalised standard deviation in phase-change memory (PCM) weight noise. A 32-bit floating point reference training (blue triangles) is included to show that optimised hardware-aware training is able to reach equivalent accuracy. It this case, the best performing model—and the one used for our weight programming optimisation inference results—was obtained using a base learning rate of 0.005, gamma learning rate decay factor of 0.95 applied once every 1.5 epochs (i.e. 2 out of 3 epochs), a dropout ratio of 0.5, and 2x the normalised PCM weight noise.

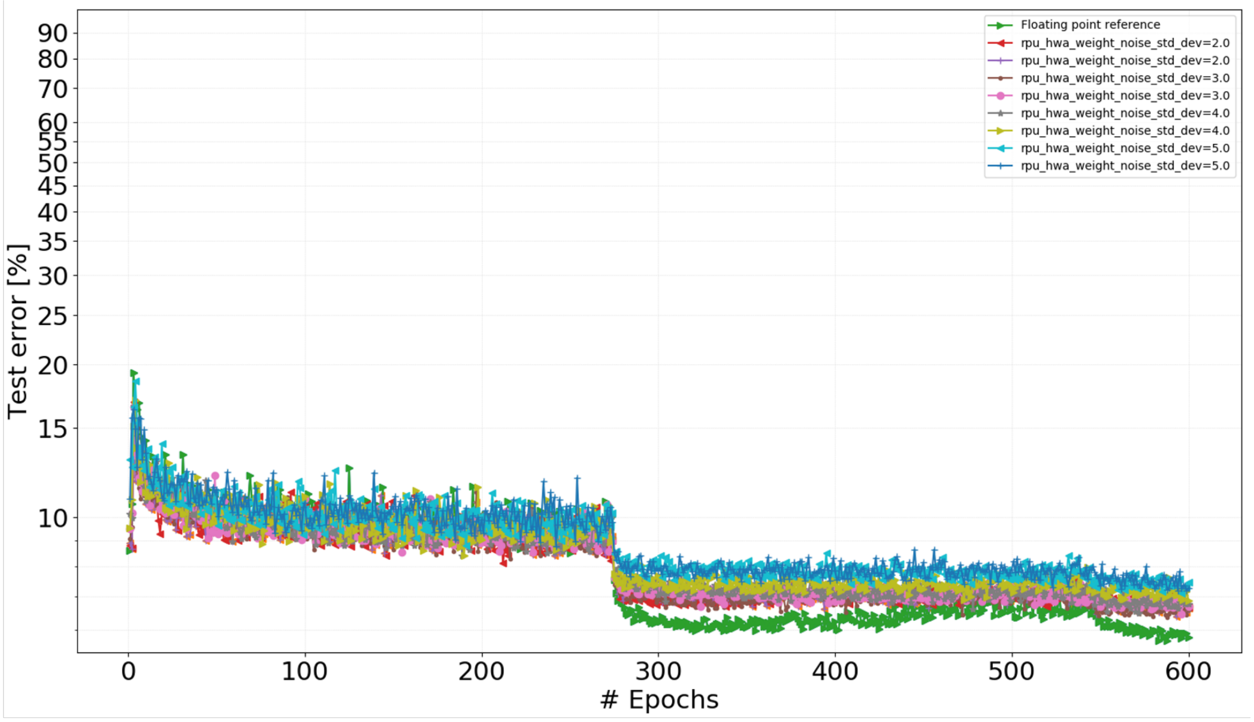

Supplementary Figure 2: Hyper-parameter scan for the hardware-aware training of the ResNet-32 network on the CIFAR-10 dataset. A 32-bit floating-point reference training (green triangles) is included to show that optimised hardware-aware training is able to reach near equivalent accuracy. Here we find using 2x the nominal PCM weight noise produces a network accuracy closest to the floating-point baseline. In this case, the learning is decayed from from 0.025 to 0.00025 over the course of training and updated using the appropriate multiplicative gamma factor every 2 epochs.

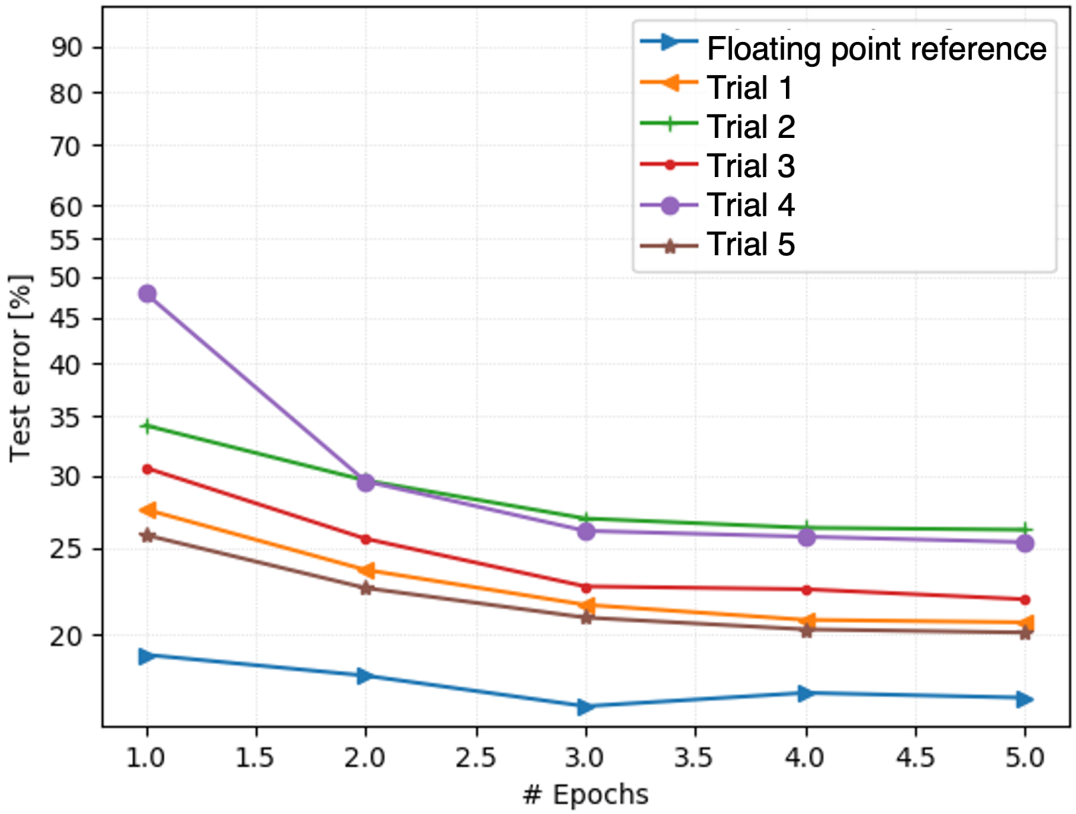

Supplementary Figure 3: Hardware-aware (HWA) training of the BERT-base (uncased) network on the General Language Understanding Evaluation (GLUE) benchmark, of which the MNLI dataset is the largest. The uncased BERT-base model in our test consists of 12 layers of transformer encoders. A 32-bit floating point reference training (blue triangles) is included to show that optimised hardware-aware training is able to reach near-equivalent accuracy. We scanned a variety of learning parameters during fine-tuning, e.g., batch size, learning rate, weight clipping, dropout, etc. We use a sequence length of 128 for better efficiency, which is sufficient for the vast majority of data samples. The learning rate is 10^{-4} (without decay) and no dropout was used. A HWA noise scale of 2.0 (twice the nominal PCM noise) produced the best training results. Five identical trials were run to account for the trial-to-trial variations. The results from the best trial (5) was used for all inference simulations reported in this work. All HWA training trials were run on NVIDIA v100 GPUs.

Impact of the Number of Discretisation Intervals on Optimal Weight Programming Strategy

The following simulation results show the impact of increasing the number of discretisation points on the weight programming strategy found by the optimisation framework.

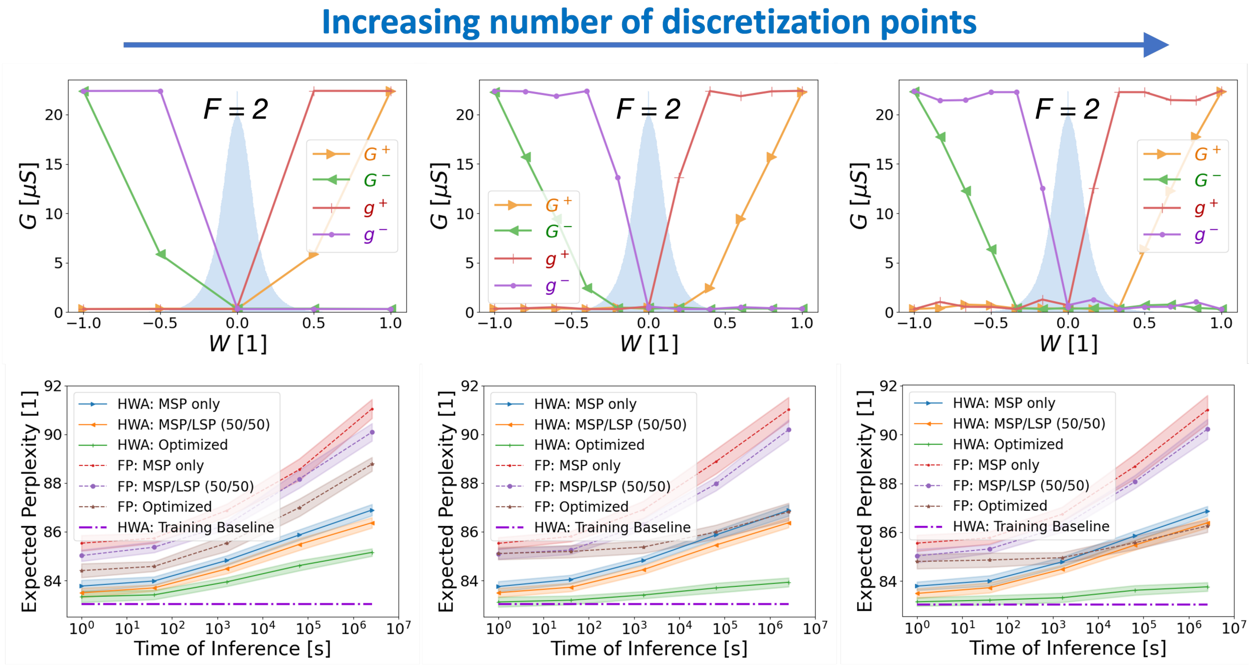

Supplementary Figure 4: Increasing the number of discretisation points allows the weight programming optimisation framework to capture more complex programming schemes, which in turn improves inference accuracy. Using only three points (left) provides a coarse outline of the optimal weight programming strategy and still improves inference accuracy over *naive* weight programming strategies. Increasing the number of points to six (center) provides additional resolution into the optimal weight programming strategy and yields further improved inference accuracy. Increasing the number of points to seven (right) produces a similar optimal weight programming strategy with little added benefit in terms of inference accuracy. This comes at increased computational expense as the dimensionality of the optimisation problem has been expanded by another four dimensions. We use six points throughout our manuscript for consistency and as a compromise between adequately capturing the optimal weight programming strategy and computational cost. The results presented here stem from the same 2-layer LSTM network within the manuscript, which was evaluated using word-based prediction on the Penn Treebank dataset. Regardless of the number of discretisation points, the optimisation framework found the optimal F factor to be two in each case.

Impact of F Factor

The main manuscript shows weight programming optimisation provides inference accuracy improvement over MSP only programming using $F = 1$, and MSP/LSP (50/50) programming using $F = 1$. Weight programming optimisation, however, finds the optimal F factor is two. Here we include simulation results for MSP only programming using $F = 2$, and MSP/LSP (67/33) programming using $F = 2$. This shows the inference accuracy benefits from weight programming optimisation are not simply from increasing F. For the MSP/LSP (67/33) case, if the weight is positive, 67% of the weight is programmed in the G^+ and 33% of the weight is in the g^+—with G^- and g^- programmed to their minimum conductance (i.e. RESET). This makes full use of the PCM dynamic range. Similarly, for negative weights, 67% is programmed in the G^- and 33% in the g^- (with G^+ and g^+ RESET).

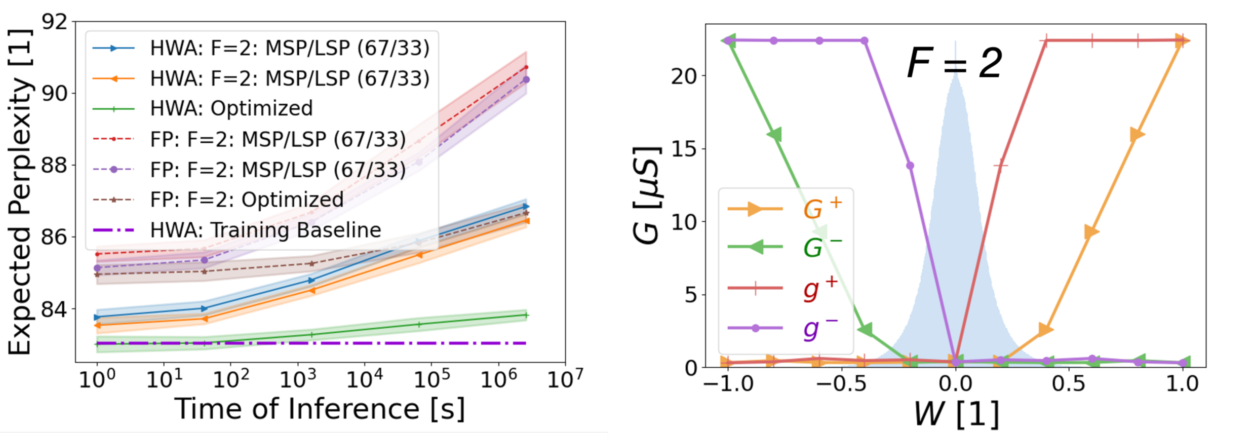

Supplementary Figure 5: Optimised weight programming strategy shows inference accuracy improvement over *naive* weight programming strategies even when increasing F in the *naive* strategies to 2 (left). Using $F = 2$ for the $naive$ strategies shows little improvement over the default $F = 1$ case. This further supports that inference accuracy improvements do not stem solely from an increase in F factor, but from the more complex weight programming schemes produced by the optimisation framework (right). The device models used in this analysis are identical to Figure 4a-c of the main text.

A Challenging Optimisation Problem

We numerically evaluate the Hessian of our error metric and find it fails the positive semi-definite test for convexity. Because the inputs (i.e. device models) to the error metric are stochastic, the error metric also becomes stochastic. In addition, this is a very high-dimensional optimisation problem (with each dimension being continuous). Even if each axis could be discretised to ten points (which it cannot), we would have $\sim 10^{26}$ possible weight programming strategies. These factors contribute to weight programming optimisation being a high-dimensional stochastic and non-convex minimisation problem.

In the following example, we provide insight into how challenging it is to find weight programming strategies that improve the error metric (and inference accuracy) beyond what can be achieved by simple MSP/LSP (50/50) weight programming, which is similar to weight slicing. We randomly sample 10 million different valid weight programming strategies from within the hypercube (using the standard 6 weight discretisation points). This brute force approach shows that of the 10 million programming strategies evaluated, the vast majority are substantially worse than our target benchmark for improvement: MSP/LSP (50/50) weight programming (vertical gray line). At best, the strategies (roughly 3 of 10 millions) are equivalent to MSP/LSP (50/50) programming. It is virtually impossible to improve the error metric to the extent shown in this work through brute force methods. We find similar difficulty using more intelligent randomised explorations of the search space using techniques like simulated annealing (i.e. basin hopping). The vertical green line represents the minimum error metric found using a well-tuned Differential Weight Evolution (DWE) approach.

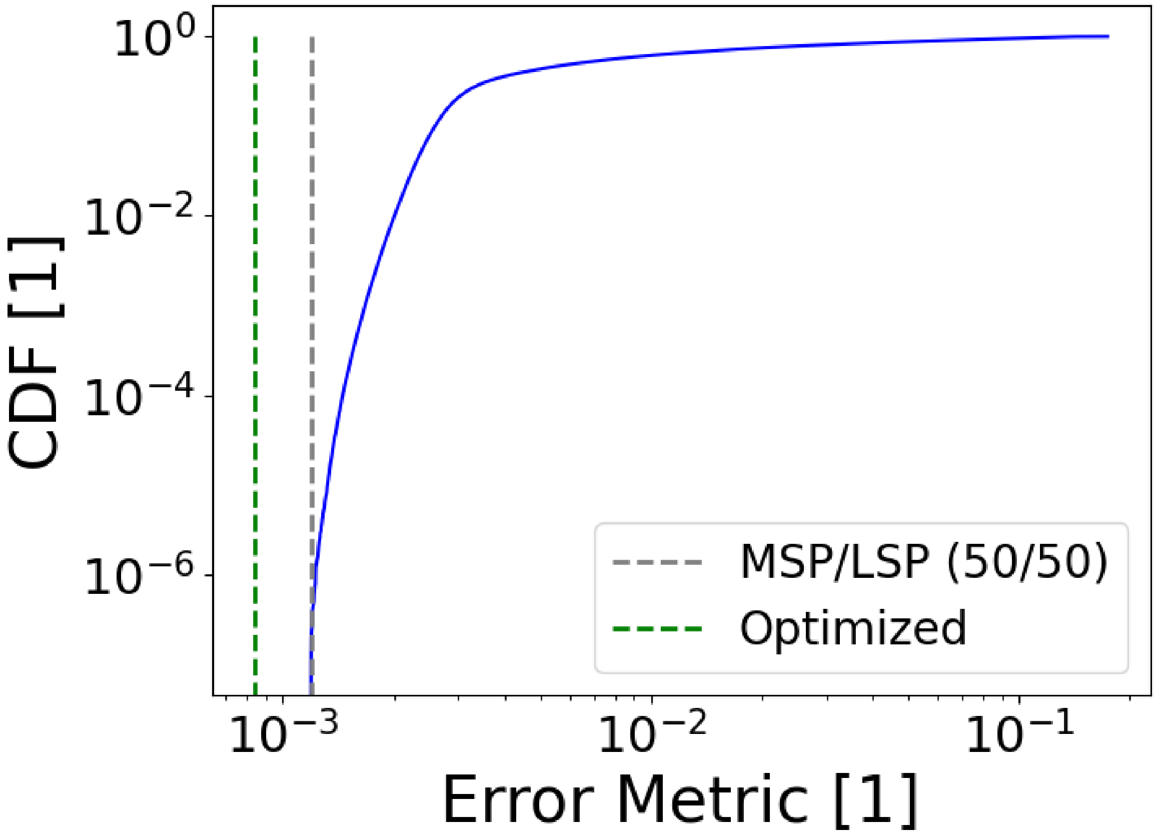

Supplementary Figure 6: Cumulative distribution function (CDF) of the error metric evaluated at 10 million randomly sampled points within the hypercube, where each point corresponds to a valid weight programming strategy. The vast majority of weight programming strategies evaluated result in substantially worse error metrics and therefore do not preserve weight fidelity well. Only 3 of 10 million samples produce an error metric that is roughly equivalent to the simple MSP/LSP (50/50) weight programming approach, which we use as our benchmark.

Hypercube de-normalisation details

Each hardware synaptic weight is defined by $W = F(G^+ - G^-) + g^+ - g^-$. During optimisation, the scale factor F is fixed to some integer value based on the hardware (e.g. 1, 2, 4). Therefore, we have four conductances. To reduce computational expense, we first note that we do not have to explore the entirety of the four-dimensional space. For instance, if we wish to program an analogue weight of 10 μS, the vast majority of potential conductance values $G^+,\ G^-,\ ,g^+$ and g^- will not sum to 10 μS. We can limit the exploration space, by realising that we actually only wish to explore a hyperplane where $W = F(G^+ - G^-) + g^+ - g^-$. In essence, g^- can be defined as $g^- = W - F(G^+ - G^-) - g^+$, which allows us to eliminate a vast majority of conductance combinations that would produce weight values no where near the desired value and limit our exploration to a three dimensional space. We can add some tolerance ΔG to allow some wiggle room around the hyperplane. This allows the weight programming optimisation to find solutions that deliberately slightly over-program or under-program certain weights to compensate for drift and achieve overall better inference accuracy over the time interval of interest.

What further complicates the problem, however, is that each G value is bounded by the previously selected G values. In our baseline conductance model, for instance, the PCM conductance range is limited to $0 - 25\mu S$. Now let's say $F = 2$ and we wish to program a weight of 75 μS. If G^+ is 25 μS, then G^- must be zero and g^+ must be at the limit of 25 μS. In this way, we see that there exist inter-dependencies between conductance values. To avoid the complexity of dealing with a long list of dependent constraints (four for each target weight) in a constrained optimisation problem, we instead opt to perform the optimisation within a hypercube, which simplifies the con-

straints, and transform those values into an eligible conductance combinations (denormalisation) in an intermediate step. This hypercube denormalisation is described by the following equations.

Hypercube Denormalisation: $Denormalise(\{x_{0j}, x_{1j}, x_{2j}, x_{3j}\}) \rightarrow \{G_j^+,\ G_j^-,\ g_j^+,\ g_j^-\}$

$$G_j^{+,min} = \text{max}((\beta_{hw}W_j - g_{max} + g_{min})/F + g_{min} - \Delta G, g_{min})$$

$$G_j^{+,max} = \text{min}((\beta_{hw}W_j - g_{min} + g_{max})/F + g_{max} + \Delta G, g_{max})$$

$$G_j^+ = \text{clip}(x_{0j}(G_j^{+,max} - G_j^{+,min}) + G_j^{+,min}, g_{min}, g_{max})$$

$$G_j^{-,min} = \text{max}(-(\beta_{hw}W_j - g_{min} + g_{max})/F + G_j^+ - \Delta G, g_{min})$$

$$G_j^{-,max} = \text{min}(-(\beta_{hw}W_j - g_{max} + g_{min})/F + G_j^+ + \Delta G, g_{max})$$

$$G_j^- = \text{clip}(x_{1j}(G_j^{-,max} - G_j^{-,min}) + G_j^{-,min}, g_{min}, g_{max})$$

$$g_j^{+,min} = \text{max}(\beta_{hw}W_j - F(G_j^+ - G_j^-) + g_{min} - \Delta G, g_{min})$$

$$g_j^{+,max} = \text{min}(\beta_{hw}W_j - F(G_j^+ - G_j^-) + g_{max} + \Delta G, g_{max})$$

$$g_j^+ = \text{clip}(x_{2j}(g_j^{+,max} - g_j^{+,min}) + g_j^{+,min}, g_{min}, g_{max})$$

$$g_j^{-,min} = \text{max}(-\beta_{hw}W_j - F(G_j^+ - G_j^-) + g_j^+ - \Delta G, g_{min})$$

$$g_j^{-,max} = \text{min}(-\beta_{hw}W_j - F(G_j^+ - G_j^-) + g_j^+ + \Delta G, g_{max})$$

$$g_j^- = \text{clip}(x_{3j}(g_{max}^- - g_{min}^-) + g_{min}^-, g_{min}, g_{max})$$

Weight Programming Optimisation Results for Various Other Device Model Combinations

Here we test weight programming optimisation on various additional device model combinations to show that it consistently improves inference accuracy for analogue memory-based DNNs. Additional results are provided for the two-layer LSTM evaluated on the Penn Treebank dataset and ResNet-32 evaluated on the CIFAR-10 dataset. Additional results are not provided for BERT-base network evaluated on the MNLI dataset due to simulation time constraints.

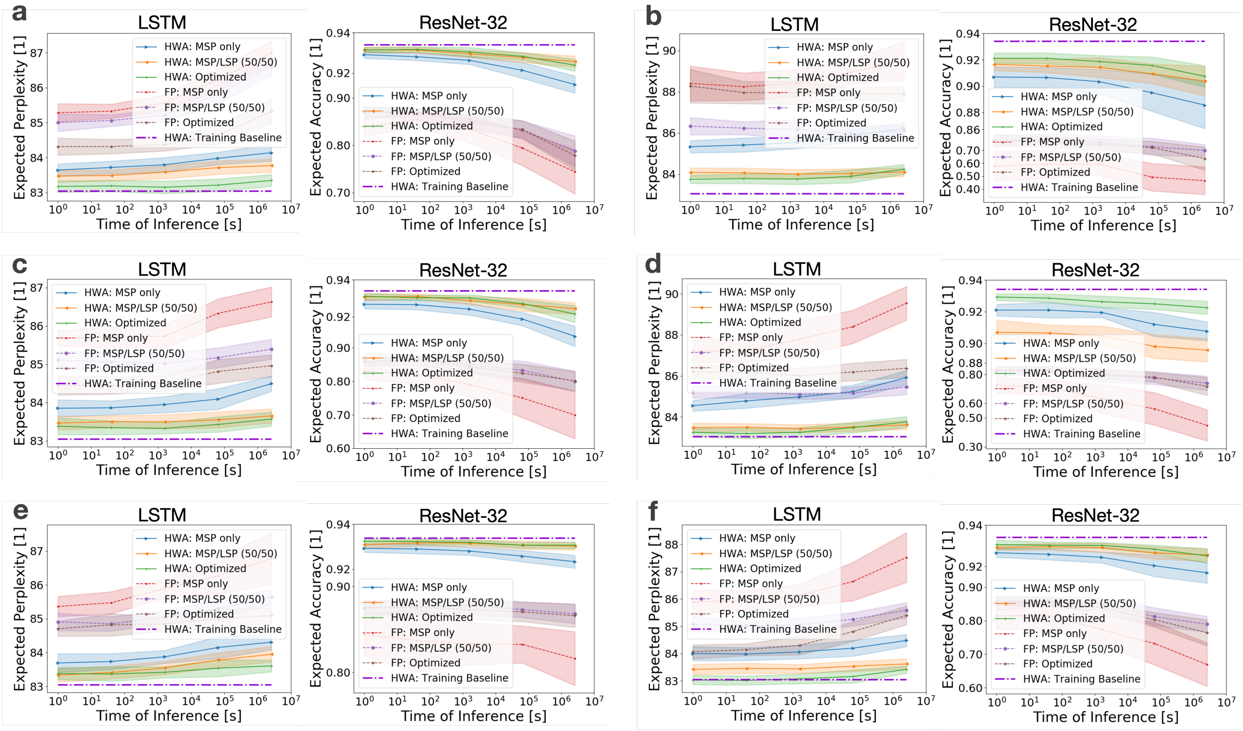

Supplementary Figure 7: Additional weight programming optimisation inference accuracy results for LSTM (Penn Treebank) and ResNet-32 (CIFAR-10) networks using various Phase-Change Memory (PCM) model combinations: a) Device I, b) Device II, c) Device III, d) Device IV, e) Device V, f) Device VI. Results show the weight programming optimisation framework consistently enhances inference performance over time and helps DNNs approach iso-accuracy. Simulations were not repeated for BERT-base on the MNLI dataset because these take considerably longer to evaluate.
